# Apigenin’s Modulation of Doxorubicin Efficacy in Breast Cancer

**DOI:** 10.3390/molecules29112603

**Published:** 2024-06-01

**Authors:** Aleksandra Golonko, Adam Jan Olichwier, Agata Szklaruk, Adam Paszko, Renata Świsłocka, Łukasz Szczerbiński, Włodzimierz Lewandowski

**Affiliations:** 1 Clinical Research Centre, Medical University of Bialystok, 15-089 Bialystok, Poland; 2 Department of Chemistry, Biology and Biotechnology, Bialystok University of Technology, 15-351 Bialystok, Poland

**Keywords:** synergistic effects, breast cancer treatment, flavonoids, nutrition, anticancer

## Abstract

Apigenin, a naturally derived flavonoid, is increasingly being acknowledged for its potential therapeutic applications, especially in oncology. This research explores apigenin’s capacity to modulate cancer cell viability, emphasizing its roles beyond its minimal antioxidant activity attributed to its basic molecular structure devoid of hydroxyl groups. We investigated apigenin’s effects on two breast cancer cell lines, estrogen-dependent MCF-7 and non-estrogen-dependent MDA-MB-231 cells. Our findings reveal that apigenin exerts a dose-dependent cytotoxic and anti-migratory impact on these cells. Interestingly, both apigenin and doxorubicin—a standard chemotherapeutic agent—induced lipid droplet accumulation in a dose-dependent manner in MDA-MB-231 cells. This phenomenon was absent in MCF-7 cells and not evident when doxorubicin and apigenin were used concurrently, suggesting distinct cellular responses to these treatments that imply that their synergistic effects might be mediated through mechanisms unrelated to lipid metabolism. A further chemoinformatics analysis indicated that apigenin and doxorubicin might interact primarily at the level of ATP-binding cassette (ABC) transporter proteins, with potential indirect influences from the AKT and MYC signaling pathways. These results highlight the importance of understanding the nuanced interactions between apigenin and conventional chemotherapeutic drugs, as they could lead to more effective strategies for cancer treatment. This study underscores apigenin’s potential as a modulator of cancer cell dynamics through mechanisms independent of its direct antioxidant effects, thereby contributing to the development of flavonoid-based adjunct therapies in cancer management.

## 1. Introduction

Breast cancer remains one of the leading causes of cancer-related deaths in women around the world. Despite advances in therapy, the complex biology of breast cancer often leads to problems with treatment efficacy and outcomes. In recent years, the integration of natural compounds into cancer therapy has attracted considerable attention, particularly to improve the efficacy of conventional chemotherapeutic agents and reduce their side effects. Among these natural compounds, Api, a flavonoid commonly found in fruits and vegetables, has emerged as an interesting compound [1].

Apigenin (Api) is known for its broad spectrum of biological activities, including anti-inflammatory, antioxidant, and anticancer properties [2]. Numerous studies have shown that it is able to inhibit cell proliferation, induce apoptosis, and inhibit angiogenesis in various cancer cell lines [3]. However, the exact mechanisms of action, particularly in breast cancer, are not yet fully understood. This is particularly important given the heterogeneous nature of breast cancer, which requires a multifaceted approach to treatment strategies [4]. 

Doxorubicin is the first-line drug in TNBC (triple-negative breast cancer) chemotherapy, currently mainly used in its liposomal form to reduce its life-threatening cardiotoxicity. Therefore, investigating its effects on the MDA-MB-231 cell line is essential as it serves as a triple-negative breast cancer (TNBC) model [5]. DOX targets the cell nucleus, intercalating into the DNA and disrupting topoisomerase II, leading to DNA damage and apoptosis via mitochondrial pathways and ROS production [5,6]. Despite its potent anticancer effects [7], DOX’s use is limited by systemic toxicity, particularly from ROS-induced oxidative stress [8]. Emerging research in nutrigenomics suggests combining DOX with phytochemicals could enhance anticancer efficacy, reduce drug dosage, and lessen side effects, presenting a promising strategy for improving chemotherapy outcomes [9,10,11].

In scientific research, flavonoids, as molecules with pleiotropic activity on cancer cells, are being investigated as potential enhancers of the action of cytostatics [12]. Due to their very limited bioavailability, researchers are considering various strategies [13], such as modifying their structure, including the creation of metal complexes, encapsulation into nanoformulations as nanoparticles [12,14], and using them as synergistic or additive substances with conventional cytostatics [15].

Recent studies indicate the potential of flavonoids such as Api to act synergistically with chemotherapeutic agents [16]. This synergy may not only increase the efficacy of the drugs but also lower the therapeutic doses required, potentially reducing the associated toxicities. In the study of Seo et al., it was demonstrated that Api influences ABC transporters by decreasing the mRNA and protein expressions of multidrug resistance 1 (MDR1) and multidrug resistance-associated proteins (MRPs) in adriamycin-resistant MCF-7/ADR breast cancer cells. This downregulation of ABC transporter expression leads to a reduced drug efflux, enhancing the accumulation of chemotherapeutic agents within the cells, thereby helping to overcome drug resistance [17]. Api’s ability to inhibit ABC transporters, despite its fewer hydroxyl groups compared to quercetin, can be attributed to specific structural features and molecular interactions. Many studies on flavonoids have focused on their capacity to reduce free radicals and interact with redox-dependent pathways. However, in the case of Api, the mechanism of action is not reliant on its antioxidant properties. Instead, Api engages directly with transport proteins through its molecular structure that allows it to bind effectively to the transporter’s active sites. This binding inhibits the transporter’s ATPase activity, which is essential for the energy-dependent efflux of chemotherapeutic drugs out of cells. Furthermore, Api affects the expression levels of these transporters. Research indicates that Api downregulates the mRNA and protein expression of key ABC transporters, like P-gp and MRP1 [17]. This modulation likely occurs through pathways independent of redox activity, potentially involving the regulation of transcription factors and signaling pathways that control transporter gene expression. This ability to alter transporter activity and expression explains how Api, despite its simple structure, effectively enhances the intracellular accumulation of drugs, improving the efficacy of treatments that are commonly pumped out by ABC transporters in resistant cancer cells [18]. Api, unlike flavonoids with a high antioxidant activity, has a favorable structure for inhibiting ABC transporters. This advantage comes from its distinct configuration of hydroxyl groups and overall molecular structure. Specifically, Api lacks hydroxyl groups at positions 3, 7, and 4′, which, as studies suggest, negatively impact the inhibition of ABCG2 transporters [19]. In contrast, the presence of a hydroxyl group at position 5 positively affects its ability to inhibit these transporters. This simpler structure without extensive hydroxylation typically found in other highly antioxidant flavonoids, like quercetin, allows Api to interact more effectively with ABC transporters, making it a promising candidate for overcoming drug resistance in cancer therapy.

Lipid droplets (LDs) in cancer cells have been implicated in various processes essential to tumor survival and progression, including energy production, redox balance, and response to stress. Furthermore, they are increasingly recognized for their role in influencing cancer cell response to chemotherapeutic agents. The review by Petan et al. (2023) and others detailed the complex interplay between lipid droplet metabolism and cancer cell viability, suggesting that a dysregulated lipid droplet turnover may present novel therapeutic opportunities [20]. Additionally, the accumulation of LDs has been associated with chemoresistance in cancer cells, indicating their potential as targets to enhance the efficacy of cancer therapies.

Therefore, this study aims to investigate the role of Api in the DOX-dependent cytotoxic effect on the breast cancer cell lines MDA-MB-231 and MCF-7. Specifically, the dose-dependent effects of Api on cell viability, its potential to enhance the efficacy of DOX, and the resulting changes in the accumulation of lipid droplets, a marker indicative of cellular stress and apoptosis, are investigated. The aim of this article is to demonstrate the impact of a simple flavonoid, which, unlike the extensively studied quercetin, lacks hydroxyl groups, with its low reactivity attributed to the absence of a hydroxyl group at position 3 of the C ring [21]. Thus, the research question posed investigates whether Api, with its low reactivity and minimal antioxidant properties, can operate through other mechanisms, independent of redox-dependent pathways. It is essential to indicate the influence on cell viability and proliferation, as well as the formation of lipid droplets in various cancer cell lines, including MDA-MB-231 and MCF-7, which are regulated by known signaling pathways, including MAPK/ERK, PI3K/AKT/mTOR, and PPARγ, in the context of lipogenesis regulation in breast cancer cells [22,23]. Understanding the interaction between Api and DOX could provide valuable insights into the development of more effective and less toxic therapeutic strategies for the treatment of breast cancer.

## 2. Results

### 2.1. Cytotoxicity Approach

#### 2.1.1. Doxorubicin Cytotoxicity

**Figure 1:**
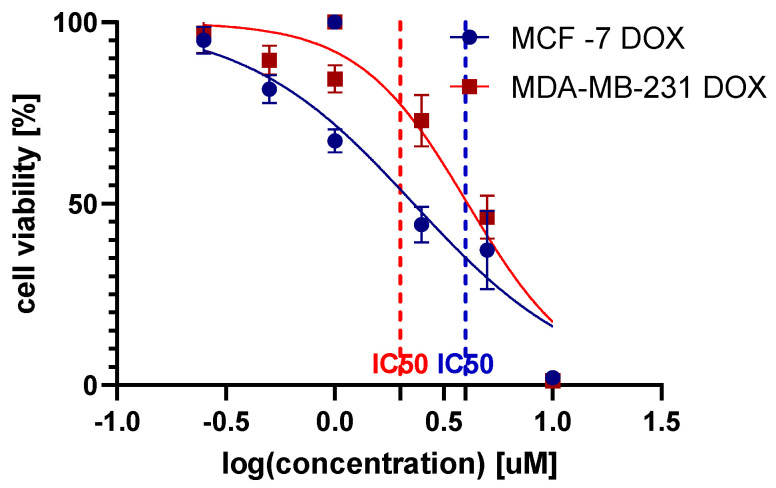
Cancer cell viability of the MCF-7 and MDA-MB-231 cell lines post-24 h of DOX exposure.

The survival curves plotted on a logarithmic concentration scale revealed the 24 h exposure effects of DOX on the MCF-7 and MDA-MB-231 cell lines. In the constructed survival curves against the log-transformed concentrations of DOX, sub-inhibitory concentrations were determined for both the MCF-7 and MDA-MB-231 cell lines (Figure 1). The IC50 values were calculated as 2.3 µM for MCF-7 DOX and 4.1 µM for MDA-MB-231 DOX, indicating a higher resistance in the MDA-MB-231 line. The goodness of fit for the dose–response curves was robust, with R-squared values of 0.89 for MCF-7 DOX and 0.90 for MDA-MB-231 DOX, reflecting a strong correlation between DOX concentration and cell viability in both cell lines.

For further studies, non-toxic concentrations were selected at half the IC50 value, specifically 1.150 µM for MCF-7 DOX and 2.036 µM for MDA-MB-231 DOX, to ensure the integrity and viability of the cells during the experimental procedures.

#### 2.1.2. Apigenin Cytotoxicity 

**Figure 2:**
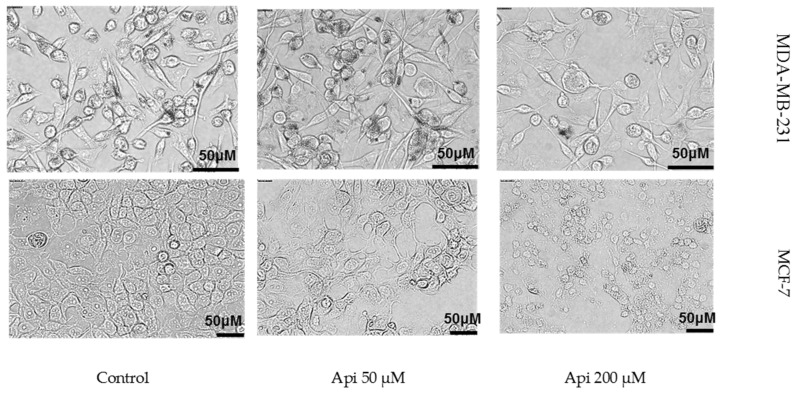
Morphological changes in the MCF-7 cell line following a 24 h incubation with Api.

At lower concentrations (12.5 µM and 25 µM), Api increased cell viability to 126.87% (±7.99) and 115.07% (±3.64) in MDA-MB-231 and MCF-7 cell lines, respectively, compared to the control. However, at higher concentrations (100 µM and 200 µM), Api significantly decreased cell viability, dropping to 58.13% (±12.33) and 32.59% (±4.51), respectively, indicating cytotoxic effects at these doses. In the MCF-7 line, Api at concentrations of 12.5 µM, 25 µM, 50 µM, 100 µM, and 200 µM resulted in mean viabilities of 102.08% (±4.42), 89.97% (±8.62), 88.57% (±2.87), 59.34% (±4.31), and 48.34% (±10.54), respectively. This contrasts with the MDA-MB-231 line, where lower Api concentrations increased cell viability, but higher concentrations (100 µM and 200 µM) led to significant viability reductions, highlighting cell line-specific differences in response to the Api treatment. Following 24 h of Api exposure, the MCF-7 cells displayed pronounced morphological changes, with a higher concentration of the compound leading to visible signs of cell death, such as cell shrinkage and fragmentation (Figure 2).

#### 2.1.3. Synergistic Effect

**Figure 3:**
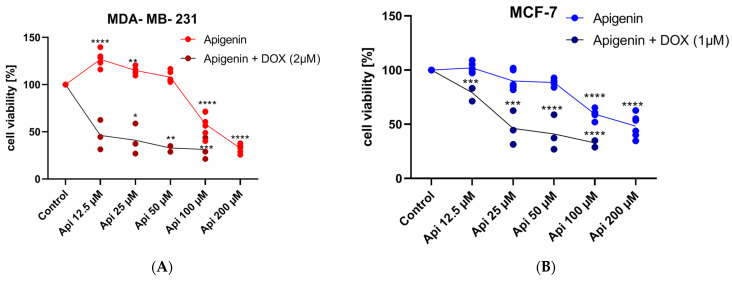
Cell viability after 24 h of DOX (IC25) and Api simultaneous treatment in MDA-MB-231 (**A**) and MCF-7 (**B**) cell lines. Significance * *p* < 0.05; ** *p* < 0.01; *** *p* < 0.0005; **** *p* < 0.0001, *n* = 3, compared to the control group.

The MDA-MB-231 cell viability in the DOX 2 µM group remained high, with a mean of 94.0% (±4.96), confirming its non-toxic nature at this concentration. Upon introducing Api in combination with DOX 2 µM, a dose-dependent decrease in cell viability was observed (Figure 3A). The mean cell viabilities for the combinations of DOX 2 µM with Api at 12.5 µM, 25 µM, 50 µM, and 100 µM were 92.59% (±8.31), 79.51% (±5.24), 77.93% (±4.57), and 66.09% (±8.98), respectively.

The statistical analysis revealed that the addition of Api, particularly at the concentrations of 25 µM, 50 µM, and 100 µM, resulted in a significant reduction in cell viability compared to the DOX 2 µM control (Figure 3A). This trend suggests a potent synergistic effect between DOX and Api, particularly at higher concentrations of Api. In the study, the MCF-7 cell line showed a statistically significant decrease in cell viability with the DOX and Api combination (Figure 3B). Specifically, the mean viability at 12.5 µM Api was 46.2% (±15.61). A dose-dependent decline in cell viability was observed across various Api concentrations combined with DOX, with mean viabilities of 41.02% (±16.21) at 25 µM, 32.84% (±3.54) at 50 µM, and 31.34% (±11.4) at 100 µM.

**Figure 4:**
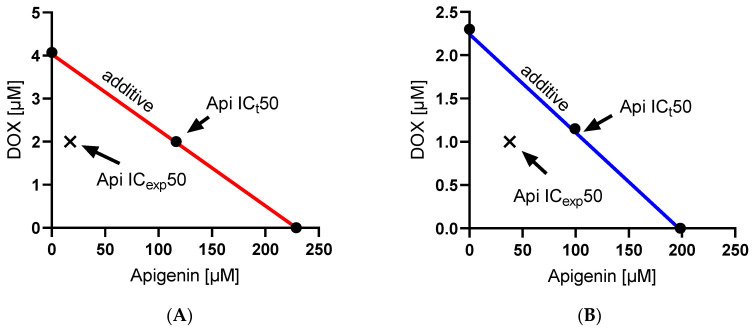
Isobologram method of determining synergy in the MDA-MB-231 (**A**) and MCF-7 cell lines (**B**).

An isobolographic analysis (Figure 4) was employed to evaluate the synergistic potential of DOX and Api combinations. The experimental findings indicate a clear synergistic effect in both cell lines as the experimental IC50 values are consistently lower than the theoretical IC50 values. For the MCF-7 cell line, the combination of Api and DOX resulted in an experimental IC50 of 37.89 µM for Api and 1 µM for doxorubicin, compared to the theoretical combination IC50 of 126.2 µM for Api and 1.15 µM for doxorubicin. This marked reduction in IC50 values from the theoretical predictions suggests a strong synergistic interaction that enhances the cytotoxicity of the drug beyond their individual effects. Similarly, in the MDA-MB-231 cell line, the experimental combination where Api was used at 17.31 µM along with 2 µM of DOX showed enhanced potency compared to the theoretical requirement of 116.47 µM Api for maintaining the effect observed with 2 µM DOX. For the MCF-7 cell line, the interaction index (γ) based on method described by Tallarida [24] was calculated as 0.626, indicating a synergistic interaction between Api and DOX since γ < 1. For the MDA-MB-231 cell line, the γ was even lower at 0.567, further supporting a synergistic interaction between the drugs in this cell line.

### 2.2. Cell Migration Speed Approach

**Figure 5:**
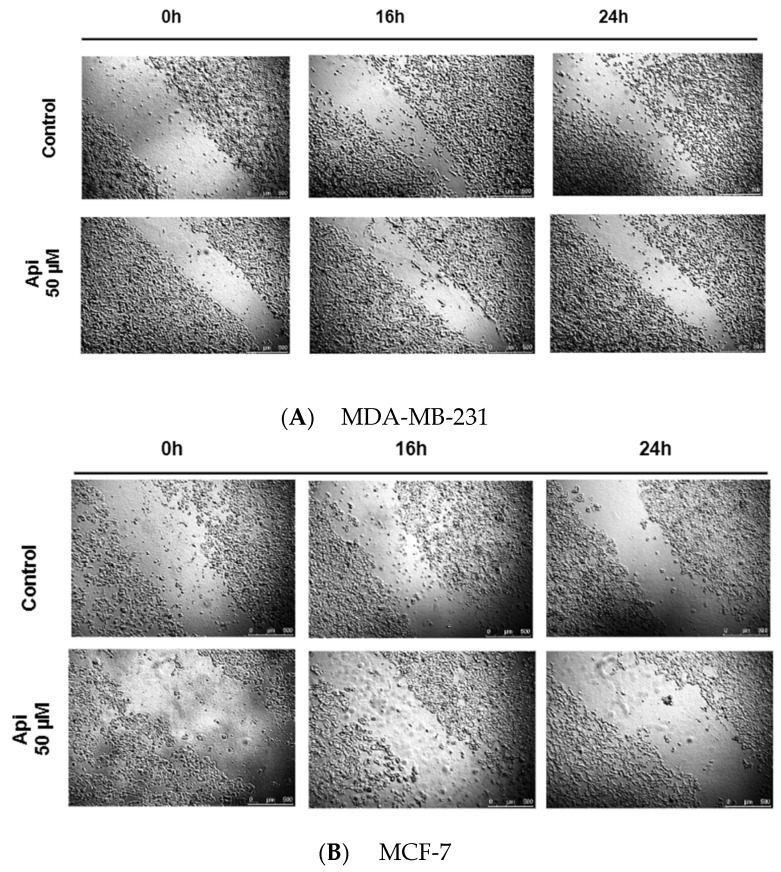
Changes in scratch width within the MDA-MB-231 (**A**) and MCF-7 (**B**) cell monolayer over time at 0, 16, and 24 h in the presence of the studied substances and without the added compound (control).

**Figure 6:**
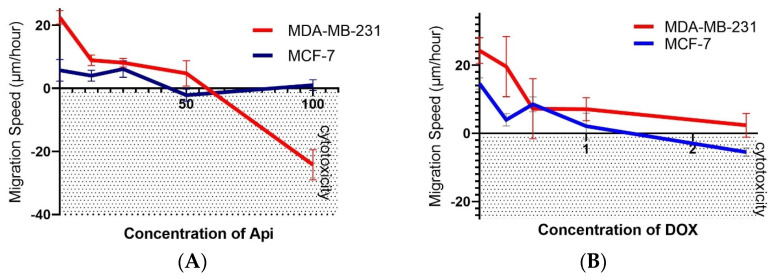
Concentration-dependent effects of Api (**A**) and DOX (**B**) on cell migration speed in the MDA-MB-231 cell line and MCF-7 cell line. Negative migration values indicate a cytotoxic effect at the corresponding concentration.

To assess the antimigration effects, it was examined whether Api, which did not demonstrate cytotoxic action on both cell lines, could influence the rate of cell migration (Figure 5A,B). Figure 6 summarizes the impact of Api (Figure 6A) and DOX (Figure 6B) on the migration speeds of the MDA-MB-231 and MCF-7 breast cancer cell lines in a wound healing assay. For the MDA-MB-231 cell line treated with Api, there was a decrease in the migration speed as the concentration increased, from 8.95 µm/h at 12.5 µM (±1.68) to 4.78 µm/h at 50.0 µM (±4.03), compared to the control group—the speed of 22.53 µm/hour (±2.12) indicates a robust migration in the absence of Api. In the MCF-7 cell line with Api treatment, the migration speed also decreased with the increase in Api concentration, from 4.04 µm/h at 12.5 µM (±1.72) to a lower speed at higher concentrations.

The average migration speed for the MDA-MB-231 cell line treated with DOX was approximately 6.92 µm/h, indicating a relatively high mobility even in the presence of anthracycline. This confirms that the MDA line may exhibit a lower sensitivity to the cytotoxic effects of DOX, retaining a significant ability to migrate despite drug exposure. On the other hand, the MCF cell line showed an average migration speed of about 3.30 µm/h, highlighting a notable reduction in cell mobility under the same conditions. Remarkably, a negative migration speed was observed at the 2.5 micromole concentration for the MCF line, which indicates a cytotoxic effect of DOX leading to the complete inhibition of cell proliferation and the retraction of the cell monolayer in the analyzed scratch area.

### 2.3. Lipid Dropplet Accumulation

**Figure 7:**
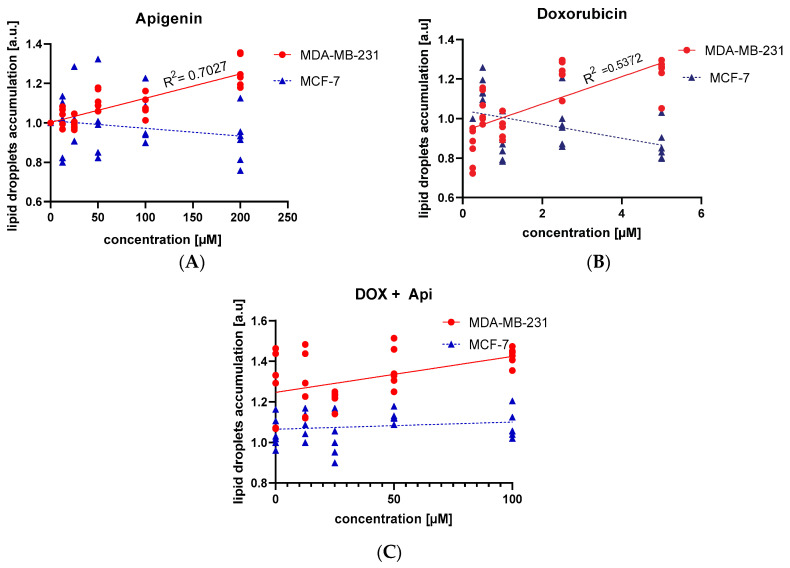
Lipid accumulation after 24 h of treatment across two cell lines treated by Api alone (**A**), DOX (**B**), or the simultaneous treatment with DOX and Api (**C**). Simple linear regression analysis between treatment concentration and lipid accumulation in the cells is shown.

In our investigation, we examined the impact of Api, DOX, and their combined application on LD formation in the MDA-MB-231 and MCF-7 breast cancer cells. Api markedly influenced LD formation in the MDA-MB-231 cells, as evidenced by a positive slope of 0.0012 and a highly significant *p*-value (<0.0001), coupled with an R^2^ of 0.70, which indicates a robust dose–response effect (Figure 7A). Conversely, in the MCF-7 cells, Api did not show a significant relationship, displaying a negative slope of −0.0004, a non-significant *p*-value (0.21), and an R² of 0.05 (Figure 7A). Regarding the DOX treatments, the MDA-MB-231 cells demonstrated a positive correlation, marked by a slope of 0.07 and a *p*-value of <0.0001 (R² = 0.54). In contrast, the MCF-7 cells showed a negative slope of −0.04 with a significant *p*-value (0.01), but a relatively low R^2^ of 0.21, suggesting a weaker correlation (Figure 7B).

The dual treatment involving Api and DOX did not significantly change these patterns in the MCF-7 cells, reinforcing the observation that Api and DOX treatments do not lead to strong correlations in LD accumulation within this cell line (Figure 7C).

### 2.4. Pathway Analysis

**Figure 8:**
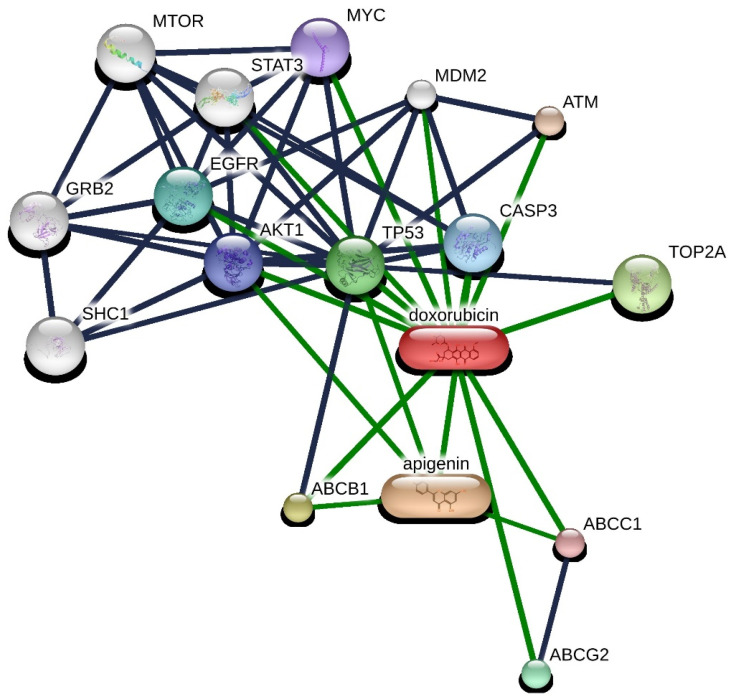
Protein–protein interaction network with DOX and Api. Interactions are represented in grey (protein–protein) and green (chemical–protein) lines, where line thickness signifies the strength of evidence. Data were derived from STITCH 5.0, with a high-confidence interaction score threshold (≥0.700).

The obtained interaction network was analyzed using STITCH 5.0. This platform facilitates the exploration of both direct (physical) and indirect (functional) associations among proteins and genes. The sources of these interactions are varied, including computational predictions, knowledge transferred across different organisms, and collated interactions from other primary databases. A list of 55 genes was generated based on 50 direct interactions (1st shell) and 5 secondary interactions (2nd shell), all filtered by a high-confidence interaction score threshold (≥0.700) (Figure 8).

Through the interaction analysis, it was demonstrated that both DOX and apigenin (Api) can directly interact with transporters, such as ABCB1, ABCG2, and ABCC1, and proteins, including T53 and MYC, with Api indirectly affecting TP53. Interestingly, AKT1 promotes the phosphorylation and inactivation of enzymes involved in lipolysis, such as hormone-sensitive lipase (HSL), leading to a decreased breakdown of lipids and an increase in lipid droplet storage [25]. Furthermore, AKT1 activates sterol regulatory element-binding proteins (SREBPs), transcription factors that enhance the expression of lipogenic genes. The activation of SREBP by AKT promotes increased lipid synthesis, contributing to lipid droplet accumulation. MYC directly enhances the transcription of genes involved in fatty acid synthesis. Key enzymes, such as acetyl-CoA carboxylase (ACC) and fatty acid synthase (FASN), are upregulated by MYC, boosting the cellular capacity to synthesize fatty acids from acetyl-CoA and malonyl-CoA, respectively [26]. Recent reports suggest that the inhibition of MYC is accompanied by intracellular lipid droplet accumulation in cancer cells as a direct consequence of mitochondrial dysfunction [26]. The interaction between Api and doxorubicin may involve complex regulatory mechanisms affecting lipid metabolism, cellular stress responses, and apoptosis, leading to non-linear effects on lipid droplet accumulation.

## 3. Discussion

Our study demonstrates that Api exhibited a dose-dependent impact on cell viability, with lower concentrations increasing viability and higher concentrations being cytotoxic. This dose-dependent behavior is consistent with the general pharmacological profile of flavonoids like Api, where their biological effects can vary significantly based on the concentration and cell type. Api, as a flavonoid, exhibits a unique dual role as both an antioxidant and a pro-oxidant, depending on various factors such as the concentration, cellular context, and oxidative stress levels [2,27,28]. At lower concentrations, Api often acts as an antioxidant; at higher concentrations or under conditions of increased oxidative stress, Api can switch to a pro-oxidant role [29].

This study suggests that Api may modulate the cancer cell environment or interact directly with DOX to enhance its cytotoxic effect. Studies have indicated that Api can modulate critical signaling pathways in cancer cells [30], such as the PI3K/PTEN/AKT pathway and the JAK/STAT [27] signaling pathway [31], which are crucial for cell survival and proliferation. In the article by Wu et al., it was observed that Api could also act as a protector against the harmful effects of DOX on non-tumor tissues. Specifically, Api was shown to mitigate DOX-induced nephrotoxicity, reducing renal injury markers and oxidative stress in normal cells without compromising the anticancer efficacy of DOX [32].

The combination of Api and DOX demonstrated a significant reduction in cell viability, suggesting a possible synergistic effect. The study also revealed that Api alone did not significantly alter LD accumulation, but its combination with DOX resulted in increased LD accumulation in the MDA-MB-231 cells. The role of LDs in cancer cells, particularly in relation to chemoresistance and cellular stress responses, is complex. The review by Petan et al. (2023) highlights the interplay between lipid droplet metabolism and cancer cell viability, suggesting that dysregulated lipid droplet turnover may present novel therapeutic opportunities [13].

The differential impact of Api and DOX on LD accumulation across the MCF-7 and MDA-MB-231 cell lines suggests a complex interplay between these compounds and the cellular mechanisms regulating lipid metabolism [33,34]. The absence of a significant LD accumulation with the Api treatment alone could indicate that Api does not directly interfere with lipid storage or mobilization pathways under the conditions tested. The distinct response of the MDA-MB-231 cells to DOX, with increased LDs and apoptotic activity at higher drug concentrations, could be indicative of a stress-related or metabolic shift towards lipid accumulation in the face of cytotoxic challenge. The slight growth of LDs at low DOX concentrations may represent a subtle cellular adaptation for survival, potentially providing energy reserves or raw materials for membrane synthesis during mild stress. The observed dose-dependent increase in LD accumulation with Api in combination with non-toxic concentrations of DOX in the MDA-MB-231 cells suggests a potential modulatory role of Api in lipid metabolism when cells are concurrently exposed to chemotherapeutic agents. The lack of change in the MCF-7 cells, except at higher Api doses, raises questions about the metabolic flexibility and adaptability of different cancer cell types. In this context, the observation that Api does not increase lipid accumulation in a concentration-dependent manner suggests that its antiproliferative action may be independent of a direct influence on lipid metabolism.

However, a full understanding of this mechanism requires further experimental research and analysis. The fact that Api does not exhibit cytotoxicity, especially at low concentrations, but shows an antiproliferative effect at just 50 µM, suggests that its anticancer activity may be mediated through mechanisms other than direct cell damage. It is possible that, at these concentrations, Api affects the regulation of the cell cycle, the induction of cell differentiation, or apoptosis mechanisms, without causing significant cytotoxicity. Possible mechanisms of the antiproliferative action of Api include the modulation of the signaling pathways responsible for cell cycle control, such as the p53, MAPK, or PI3K/Akt pathways, the inhibition of cyclin-dependent kinases, or direct influence on transcription factors controlling the expression of genes responsible for cell proliferation [31,35,36]. Research has highlighted Api’s role in disrupting the normal progression of the cell cycle, specifically by causing cells to pause at the G2/M phase. This disruption is linked to the suppression of specific kinases like p34(cdc2), which play pivotal roles in cell cycle progression, alongside a decreased presence of proteins such as p34(cdc2) and cyclin B1 [37,38].

The differential impact of Api and DOX on LD accumulation across the MCF-7 and MDA-MB-231 cell lines suggests a complex interplay between these compounds and the cellular mechanisms regulating lipid metabolism. The absence of a significant LD accumulation with the Api treatment alone could indicate that Api does not directly interfere with lipid storage or mobilization pathways under the conditions tested. The distinct response of the MDA-MB-231 cells to DOX, with increased LDs and apoptotic activity at higher drug concentrations, could be indicative of a stress-related or metabolic shift towards lipid accumulation in the face of a cytotoxic challenge.

The potential interaction between Api and DOX arises from their complementary mechanisms of action. Api, known for its ability to induce cell cycle arrest in the G2/M and G1 phases, could theoretically enhance the efficacy of DOX by causing a buildup of cells at specific checkpoints and may increase the population of cells in the phases where DOX is most effective (its anticancer effects are most potent during the G2 and S phases of the cell cycle). The lack of changes in the content of lipid droplets in a concentration-dependent manner does not necessarily indicate the absence of Api’s effect through the impact on energy pathways. However, this topic requires very thorough research involving the analysis of antioxidant capacity content and the expression of genes for antioxidant enzymes [39,40]. Interestingly, the study also highlighted that, while Api induced lipid droplet formation in a dose-dependent manner in the MDA-MB-231 cells, this effect was not observed when both compounds were used together, suggesting that the primary interactions between these compounds might involve mechanisms unrelated to lipid metabolism.

The investigation into the molecular underpinnings of DOX and Api effects on cellular processes has yielded a comprehensive protein–protein interaction network, as presented in Figure 8. The robust analysis using STITCH 5.0 delineated a network of 55 genes interconnected through 50 direct and 5 secondary associations, predicated on a high-confidence interaction threshold. The resulting network, incorporating key signaling molecules, such as MAPK8, PTEN, and TP53, lays a foundation for understanding the complex regulatory mechanisms influenced by these compounds. The enrichment of pathways related to oxidative stress and the regulation of cell death, including apoptotic processes, corresponds to the known pharmacodynamics of DOX and Api [41]. DOX proclivity to induce oxidative stress, leading to apoptosis, is well-documented, and the identified enrichment corroborates this mode of action at the genetic interaction level [42]. Numerous studies have proven that Api is an effective inhibitor of ABC transporters, and there is also evidence of an interaction between this phenolic compound and the AKT-dependent pathway. This interaction with the AKT pathway may enhance Api’s ability to modulate cellular responses, potentially augmenting the therapeutic efficacy of doxorubicin through a synergistic effect [41]. By inhibiting ABC transporters and affecting the AKT pathway, Api could alter drug efflux and cellular survival mechanisms, thereby enhancing the cytotoxic impact of doxorubicin on cancer cells.

The flavonoid structure of Api, particularly the C2–C3 double bond, has been linked to anticancer activity due to its ability to inhibit membrane efflux transporters in resistant breast cancer cells. Api’s effectiveness is also attributed to certain structural features, such as the OH group in C-5 and the O–CH3 group in C-3, which are thought to have inhibitory activity against cancer resistance proteins [43]. Furthermore, Api has been reported to enhance chemotherapy-induced apoptosis by modulating the expression levels of mitochondrial proteins. This synergistic effect with chemotherapy drugs such as DOX has been demonstrated in several cancer cell lines [41], supporting the use of Api as a chemosensitizer. For instance, Api has been shown to upregulate pro-apoptotic proteins and downregulate anti-apoptotic proteins, contributing to the induction of both intrinsic and extrinsic apoptosis pathways in cancer cells [4].

## 4. Materials and Methods

Cell Culture: The human breast cancer cell lines MDA-MB-231 and MCF-7, acquired from ATCC, were cultured in Dulbecco’s modified eagle medium (DMEM) with high glucose (Thermo Fisher Scientific, Waltham, MA, USA, Cat. No. 11965092) and 1% streptomycin–penicillin (strep-pen). The medium was further supplemented with 10% fetal bovine serum (FBS), 100 U/mL penicillin, and 100 µg/mL streptomycin. The cells were incubated at 37 °C in a humidified atmosphere with 5% CO_2_. The specific brands and catalog numbers for FBS and penicillin–streptomycin were not identified as they are commonly available from multiple suppliers.

Cell Viability Assay: The MDA-MB-231 and MCF-7 cells were seeded in 24-well plates twice with fresh medium (Sigma DMEM, 10% FBS, PenStrep) at densities of 1.5 × 10^5^ (MDA-MB-231) and 2 × 10^5^ (MCF-7) cells, respectively. The density was experimentally determined to achieve 70% confluency after overnight incubation. The cultures were established in T-75 flasks (for passaging) with 13 mL of the medium for the further propagation of the lines. Viability was measured using the CellTiter-Blue reagent (Promega, Cat. Nos. G8080 for 20 mL and G8081 for 100 mL), which changes color due to the conversion of resazurin to resorufin by live cells. The cytostatic effect of ligands was evaluated within a concentration ranging from 12.5 to 200 µM. Concentrations above 200 µM were not used due to potential toxicity, pro-oxidative actions, and solubility limitations. The inhibition potency was assessed using a variable slope dose–response model to analyze the effects of inhibitor concentrations, which were logged against normalized responses for each cell line. The dataset included 42 logged inhibitor concentration points, with 28 responses measured in MCF-7 and 34 in MDA-MB-231. The IC50 values were determined as 2.30 for MCF-7 (R^2^ = 0.886) and 4.07 for MDA-MB-231 (R^2^ = 0.9063). The formula IC25 = IC50 × ((100 − 25)/25)^(1/HillSlope) was used to calculate the concentrations required to achieve 25% inhibition in both cell lines. The IC50 value for Api was determined using a dose–response curve, where the inhibitor concentration was plotted against the normalized response. The analysis was carried out by fitting the experimental data to a sigmoidal curve using non-linear regression, which allowed the estimation of the best-fit values for the IC50 and its logarithmic transformation (logIC50).

Drug Interaction Calculation: For each drug or drug combination, the dose necessary to achieve 50% of the maximum possible effect (IC50) was calculated. To assess the interaction between drugs, an isobolographic analysis was conducted along with the calculation of interaction indices according to the method described by Tallarida et al. [24]. Differences were considered statistically significant at a *p*-value ≤ 0.05. The interaction index (γ) is calculated using the following formula: γ = a/A + b/B, where A and B are the doses of the drugs administered alone that induced a 50% cell viability, a and b are the concentrations of the drugs administered together that produced the same cytotoxic effect. An isobolographic analysis is a graphical interpretation of drug interactions. An isobologram was plotted by marking the doses of Api and DOX on the axes, which alone produced a specified effect, like 50% of the maximum possible cytotoxic effect. The line connecting these points represents the line of additive effect. The coordinates of all points on this line correspond to the combined doses of the drugs that produce the expected analgesic effect assuming an additive interaction (point X). Coordinates of all points below this line correspond to the doses of substances that, when combined, produce the expected cytotoxic effect assuming a synergistic interaction between them.

Wound Healing Assay: Cells from both the MDA-MB-231 and MCF-7 cell lines were cultured in 6-well plates until they reached 90% confluency. At this point, a sterile 200 µL polystyrene pipette tip was used to create a scratch wound. The medium was carefully replaced with fresh medium following the scratch. Subsequently, each well received 3 mL of either the control medium or a medium containing Api dissolved in DMSO and diluted in PBS. The target concentrations of the compound—12.5, 25, 50, 100, and 200 µM—were prepared by dilution in the freshly made culture medium. Immediately after the addition of the substances, photographs were taken to measure the width of the scratch using Leica LAS X Life Science Microscope Software Ver No 4.13. The migration speed of cells at 8, 16, and 24 h was calculated using the formula VT = (D_0_ − DT)/T, where VT represents the migration speed at time T_0_, D_0_ is the initial average distance between the cells, DT is the average distance at time T, and T is the elapsed time in hours, allowing for a precise quantification of cell migration dynamics over specified intervals.

Lipid Droplet Staining: Cells were triple washed with cold PBS, fixed with 10% formalin for one hour, and then treated with 60% isopropanol. Lipid droplets were stained using a solution of Oil Red O (Merck KGaA, Darmstadt, Germany, Cat. No. O1516 Sigma-Aldrich, St. Louis, MO, USA), prepared by dissolving 0.3 g of Oil Red O powder in 60% isopropanol. Staining was conducted for 15 min at room temperature, followed by washing with 60% isopropanol and distilled water. The quantitative assessment of Oil Red O content was performed by measuring the absorbance at 518 nm. The normalized lipid droplet content was calculated using the formula: Normalized LD Content = (Absorbance at 518 nm (Sample)/Absorbance at 518 nm (Control)) × (Percent Live Cells in Sample (CTB)/Percent Live Cells in Control (CTB)).

Preparation of Drug Concentrations: Various concentrations of the compounds were prepared for determining the IC50 values and assessing cell viability in their presence. The control cells were exposed to 1% DMSO. The control cells were exposed to 1% DMSO. Apigenin (Sigma Aldrich, CAS: 520-36-5) was dissolved in DMSO to a stock concentration of 0.04 M and then added to the culture medium either alone or with doxorubicin hydrochloride (Dox-HCl), purchased from Sigma (CAS: 25316-40-9), in appropriate volumes to ensure that the DMSO did not exceed 0.1% of the solution volume. All experiments were conducted in triplicate and repeated three times to ensure the reproducibility and reliability of the results.

Molecular interaction and pathways analysis: Interaction networks for doxorubicin and apigenin were constructed and analyzed using the STITCH database (http://stitch.embl.de/, accessed on 27 March 2024), setting a high confidence threshold at 0.7 for Homo sapiens. The visualization parameters were constrained to 50 primary interactors and 5 secondary interactors to maintain analytical clarity.

Statistical Analysis: Statistical analysis and data visualization were conducted using the GraphPad Prism software (version 9.4.1 for Windows, GraphPad Software, Inc., La Jolla, CA, USA, www.graphpad.com, accessed on February 2022). Data were presented as mean values ± standard deviation (SD) from at least three independent experiments, each performed in triplicate. A one-way analysis of variance (ANOVA) was used for multiple group comparisons, with post hoc tests applied as appropriate. Differences were considered statistically significant at *p* < 0.05.

## 5. Limitations and Future Perspectives

In this study, we focused on identifying the potential synergistic effect of a flavonoid and a cytotoxic drug on two cancer cell lines. Api, as a flavonoid with a well-researched safety profile, has been previously described in terms of its effects on both cancerous and non-transformed cell lines. The observed interaction is very promising and has directed our future research towards studying the impact of Api and DOX on mitochondrial bioenergetics, gene expression involved in apoptosis, and their roles in cytotoxic effects. This research will extend to non-transformed cell lines, including fibroblasts and cardiomyocytes, allowing for a comprehensive description of the action profile under in vitro conditions.

## Data Availability

The original contributions presented in the study are included in the article, further inquiries can be directed to the corresponding authors.
